# Meningiomas and Somatostatin Analogs: A Systematic Scoping Review on Current Insights and Future Perspectives

**DOI:** 10.3390/ijms24054793

**Published:** 2023-03-01

**Authors:** Sofie Eline Tollefsen, Ole Solheim, Patricia Mjønes, Sverre Helge Torp

**Affiliations:** 1 Department of Clinical and Molecular Medicine, Faculty of Medicine and Health Sciences, Norwegian University of Science and Technology (NTNU), 7491 Trondheim, Norway; 2 Department of Neurosurgery, St. Olavs Hospital, Trondheim University Hospital, 7030 Trondheim, Norway; 3 Department of Neuromedicine and Movement Science, Norwegian University of Science and Technology, 7491 Trondheim, Norway; 4 Department of Pathology, St. Olavs Hospital, Trondheim University Hospital, 7006 Trondheim, Norway

**Keywords:** brain tumor, meningioma, somatostatin, somatostatin receptor, somatostatin analog, octreotide, pasireotide, lanreotide, treatment, therapy

## Abstract

Meningioma is the most frequent brain tumor, and the incidence is ever-increasing. Though often benign and slow growth, recurrence rates are substantial and today’s surgical and radiation-based treatment are not without complications. No drugs specific for meningiomas are hitherto approved and patients with inoperable or recurrent meningioma are left with few treatment options. Somatostatin receptors are previously detected in meningiomas and may inhibit growth when stimulated by somatostatin. Hence, somatostatin analogs could provide a targeted drug therapy. The aim of this study was to compile the current insights of somatostatin analogs for patients with meningioma. This paper adheres to the PRISMA extension for Scoping Reviews. A systematic search was conducted in the search databases PubMed, Embase via Ovid, and Web of Science. Seventeen papers adhered to the inclusion and exclusion criteria, and critical appraisal was conducted. The overall quality of evidence is low, as none of the studies were randomized or controlled. Various efficacy of somatostatin analogs is reported, and adverse effects are sparse. Due to the beneficial effects reported by some studies, somatostatin analogs may offer a novel last-option treatment for severely ill-patients. Nonetheless, only a controlled study, preferably a randomized clinical trial, could clarify the efficacy of somatostatin analogs.

## 1. Introduction

Meningioma is the most frequently occurring tumor in the central nervous system [1] and incidence rates are rising, presumably much due to increased use of magnetic resonance imaging (MRI) [2,3]. The tumors are most often benign and slow-growing, and patients may live with the disease for decades without noticing any symptoms [4]. According to the Central Brain Tumor Registry of the United States (CBTRUS), the incidence rates for meningiomas is 9.51 per 100,000 population [5]. However, based on incidental findings after MRI scans, meningiomas may have a suggested prevalence of 1% in the adult population [2]. Meningiomas are classified according to the Central Nervous System World Health Organization (CNS WHO) 2021 classification, which is the first edition to also include diagnostic molecular pathology [6]. There are 15 subtypes of meningiomas and three CNS WHO grades, with benign CNS WHO grade 1 being the most frequent, accounting for 80.1% of all meningiomas [5,6].

Surgery is the primary treatment for most patients suffering from growing or symptomatic meningioma [7]. However, tumors involving important neurovascular structures, engulfing cranial nerves, exhibiting extensive intraosseous growth, or widespread or multifocal dural involvement can be difficult to resect [8,9], and severe complications may occur [10]. Radiotherapy can be an attractive alternative with good tumor control rates for smaller tumors, although complications can be seen [3,4,10,11]. Due to the increase in incidental findings of small asymptomatic meningiomas, active surveillance is increasingly used for these patients [10]. Despite often benign histology, recurrence rates after treatment are still substantial, not at least in CNS WHO grade 2 and 3 meningiomas [10,12]. As no drugs specific for meningiomas are approved by the United States Food and Drug Administration (FDA), patients are left with few treatment options except reoperations, irradiation or re-irradiation in cases of recurrent or anaplastic meningiomas [13]. Accordingly, establishment of pharmaceuticals would be an important step towards improved patient care.

Somatostatin, a potent inhibitor that binds to somatostatin receptors (SSTRs), contributes to the regulation of tumor growth [14]. The membrane-bound G-protein coupled SSTRs use guanosine triphosphate (GTP) to trigger intracellular pathways. This leads to inhibition of adenylyl cyclase, activation of phosphotyrosine phosphatase (PTP) and modulation of mitogen-activated protein kinase (MAPK). Following this, cell cycle arrest is induced [15,16,17,18], illustrating the potential anti-proliferative effect of SSTRs in meningioma cells. Antitumoral effect may also be generated by activation of SSTRs on normal cells, as this may induce vasoconstriction, decreased secretion of growth factors, modulation of immune cell function and inhibition of angiogenesis, providing an antitumoral effect [15,16,18].

Hence, the discovery of SSTRs in meningiomas led to prospering hope of targeted drug therapy [19,20,21]. Due to the potent effect and short half time of somatostatin, synthetic somatostatin analogs were developed for drug trials [22,23]; with octreotide, pasireotide and lanreotide as the most prominent. As of today, the European Association of Neuro-Oncology (EANO) has not provided guidelines on the use of somatostatin analogs for meningiomas [7,24], and Norwegian guidelines consider somatostatin analogs as experimental treatment [25].

In this present systematic scoping review, we sought to address what is currently known and unknown about the potential for treatment of meningiomas with somatostatin analogs.

## 2. Materials and Methods

This paper adheres to the PRISMA extension for Scoping Reviews (PRISMA-ScR) [26] and searched for all relevant peer-reviewed journal papers with no restrictions regarding language or publication date. The protocol for this systematic scoping review is not published.

The following three search databases were used: PubMed, Embase via Ovid, and Web of Science. Two search concepts were applied: (a) meningioma and (b) somatostatin analogs. MeSH terms, Emtree terms and a free text search were utilized. Somatostatin analogs are known by several synonyms, including somatostatin receptor agonists, and search concept (b) also contained the generic names of the most relevant somatostatin analogs, e.g., octreotide, pasireotide, lanreotide and angiopeptin. Sandostatin^®^ is a common brand name for octreotide and were therefore included in search concept (b). Identified MeSH terms and Emtree terms were applied for all somatostatin analogs. After combining relevant search terms for each search concept using the search operator OR, the two search concepts were combined using the search operator AND. The search was last updated on 11 November 2022. See Supplementary File S1 for detailed search history.

Inclusion criteria for this systematic scoping review were (1) meningioma and (2) systemic treatment with a somatostatin analog. Exclusion criteria were (i) patients < 18 years old and (ii) studies on imaging or scintigraphy alone. All secondary research was excluded, including editorials, reviews, and commentaries. In vitro studies establish the knowledge foundation for later clinical studies and were therefore not excluded.

After completing the searches, all obtained records were uploaded to the reference manager EndNote X9.2. All records were screened by title and evaluated for further inclusion adherent to the inclusion and exclusion criteria. Remaining records were then screened for inclusion by abstracts. Thereafter, full text papers were accessed for all remaining records before final assessment for inclusion to this systematic scoping review. For all included in vivo studies, the following data was registered: number of patients, CNS WHO grade and/or subtype, treatment received, clinical response, radiological response, the presence of SSTRs, and adverse effects. If stated, progression-free-survival at six months (PFS-6) was contained. Regarding in vitro studies, the aim of the study and a summary of the results were retrieved. The inclusion of papers and data charting were conducted by a medical research student (SET).

Critical appraisal tools are essential to assess the quality of research. For this systematic scoping review, validated checklists for critical appraisal from the Joanna Briggs Institute (JBI) of The University of Adelaide, Australia, were used. JBI has designed their checklist according to study design and the following checklist were applied: “checklist for case reports” and “checklist for quasi-experimental studies” [27].

## 3. Results and Discussion

### 3.1. The Included Papers

**Figure 1:**
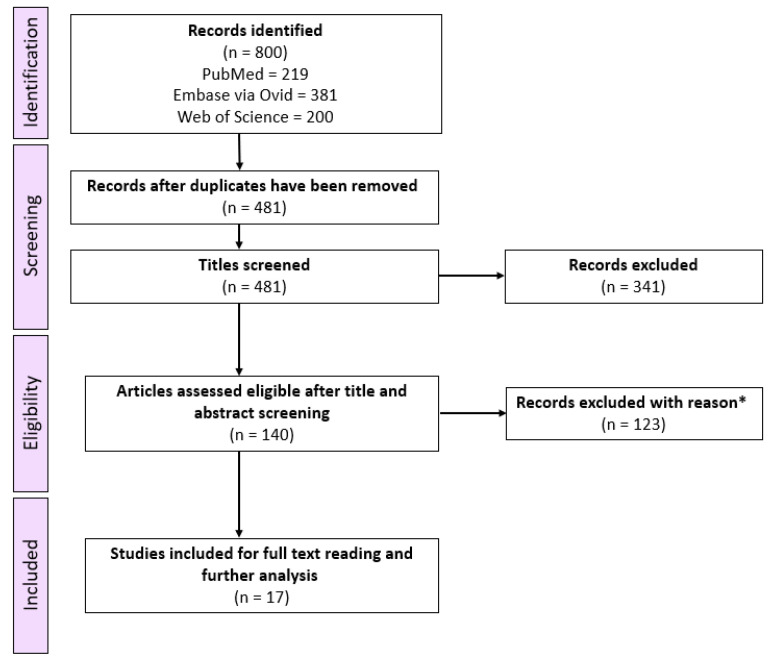
PRISMA flow chart for the selection of papers included in this systematic scoping review. * Excluded records based on the inclusion and exclusion criteria.

A PRISMA flow chart describing the selection of papers is presented in Figure 1. As seen, the systematic search identified a total of 800 records, of which 319 duplicates were removed. After reviewing all records by title, an additional 341 records were excluded following the inclusion and exclusion criteria. The remaining 140 records were screened by abstract resulting in the exclusion of another 123 records. Finally, a total of 17 studies were included in this systematic scoping review. All retrieved articles were written in English.

### 3.2. Characteristics of Sources of Evidence

Among the 17 reviewed studies, there were five case reports [28,29,30,31,32], three retrospective case studies [33,34,35], five prospective studies [36,37,38,39,40], and four in vitro investigations [41,42,43,44]. In this systematic scoping review, the diverse effect of somatostatin analogs in the clinical treatment of meningiomas are presented. Some studies report favorable results following treatment [28,30,31,36,40], while others report no response or disease progression after treatment [34,37,39]. Further on, everolimus combined with octreotide or pasireotide were found favorable [33,35,41,42].

### 3.3. Summary of Sources

A total of 129 patients were included in the 13 in vivo studies, of which 97 were included in prospective studies. Of the 129 patients, 115 (89.1%) had previously been operated and 96 (74.4%) patients had undergone radiotherapy. Only three (2.3%) patients were previously untreated. Data on previous treatments were missing for nine patients. An overview of the 17 included papers is found in Table 1 and Table 2.

While Graillon et al. reported an octreotide dose-dependent inhibition of cell viability in vitro [43], Koper et al. observed a significant growth in cultured meningioma cells following exposure to octreotide [44]. The same discrepancy of effect was reported in the in vivo studies. While three prospective studies and one case report found no radiological and only sparse clinical effects following octreotide treatment [29,36,37,39], two case reports described clinical remission in their two patients after treatment [30,31]. An in vitro investigation found pasireotide to be a significantly better inhibitor of cell viability, when compared to octreotide [42]. In vivo, pasireotide was reported to be well tolerated by patients, but was unsuccessful in obtaining a radiological response [38]. In this systematic scoping review, we found no in vitro studies on lanreotide. Nevertheless, one clinical case report describes a radiological response, with a decrease of tumor volume by 35%, and progression free survival for more than two years following lanreotide treatment in a patient with progressing meningioma after multiple treatments [28].

In the identified studies, various endpoints were utilized to measure response to treatment. Several studies applied progression free survival as endpoint [33,35,36,38,39]. PFS-6 ranged from 17 to 60% in the included papers, with a median of 47.2%. Radiologic response was used as endpoint in several studies [36,37,38,39]. Chamberlain et al. reported a partial radiologic response (PR) in 31% of patients, with PR defined as >50% reduction in tumor size on consecutive MRI scans at least two months apart, with no increase in the patients’ neurological symptoms or need for corticosteroids [36]. However, Johnsen et al. did not observe any radiologic response, using similar criteria as Chamberlain et al, with partial response defined as a decrease in tumor size of >50% [37]. Simó et al. used radiological partial response, defined as decrease of ≥50% in two-dimensional maximum diameters, but no radiological partial responses were observed [39]. Neither Norden et al. found a radiological response following treatment, applying the modified MacDonald criteria [38].

Graillon et al. and Furtner et al. reported tumor growth rates as their endpoint. Graillon et al. found a major decrease, defined as >50% reduction in growth rate assessed at three months in 78% of the tumors [40], while Furtner et al. only found a slight reduction in tumor growth rate following somatostatin analog treatment [34].

### 3.4. Critical Appraisal within Sources of Evidence

All included papers were evaluated for critical appraisal according to JBI checklists for case reports and quasi-experimental studies. As for the clinical quasi-experimental studies, similarity within patient cohorts is inadequate as the patients differed in factors such as CNS WHO grade, previous treatment, Karnofsky status and treating hospital. None of studies included control groups. Most studies presented multiple outcome measures, and most commonly including both survival analyses and radiological response. Incomplete follow-ups were accounted for in all papers. The absence of randomization and control groups constitute a potential bias in all included clinical studies, and evaluation of potential effects on both survival and progression free survival is difficult. For the retrospective studies, inclusion of patients and selection of treatment was conducted by the treating oncologists, and there was no clear standardization in treatment (dosage and duration), imaging protocols, image intervals, or clinical management algorithms. This may cause latent bias as several non-controlled variables were not explored in the studies. Also, assessments of progression free survival will potentially be affected by image intervals and completeness of clinical documentation. All clinical case reports have adequate descriptions of demographics, patient pathways, clinical conditions, diagnostic tests, and interventions. The post-intervention condition of the patients and any adverse effects were clearly stated. Still, classification of adverse drug reactions was not standardized. Overall, the included quasi-experimental studies were considered of low quality with high risk for biases and high risk of confounding factors. Many of the included patients had previously been unsuccessfully treated with radiotherapy, but both temporary tumor-swelling and the late growth inhibition after radiotherapy may hamper causal interference of effects of subsequent treatment. Further, quantitative image assessment in post-treatment scans can be hampered by a number of other factors, including contrast enhancing scarring or enhancing post-surgical peritumoral infarctions, or ill-defined intraosseous or intravenous growth. Also, assessing progression or response above or below a certain percentage in irregular shaped tumors with some variation in image protocols is notoriously difficult. Perhaps most important, radiological assessment was not blinded in any of the studies.

### 3.5. The Molecular Mechanisms of Somatostatin Analogs

Somatostatin analogs bind to SSTRs with various affinity. Octreotide is known for its higher affinity to SSTR2 and SSTR5 [45], while pasireotide favors SSTR1, SSTR3 and SSTR5 [38]. Lanreotide binds to SSTR5, but mainly to SSTR2 [46]. Even though SSTR2 is established as present in most meningiomas, the distribution of the other SSTRs differs between publications [20,21,47,48]. Hence, identification of different SSTRs within the tumor biology could help decide on the most efficient somatostatin analog and influence the treatment response. Many studies have used OctreoScan, a radiolabeled octreotide scintigraphy, to decide on the presence of SSTRs in advance to treatment. Yet, as the epitope octreotide mainly binds to SSTR2 and the distribution of other SSTRs is not mapped, OctreoScan may not be sufficient to predict treatment response [36]. Also, DOTA-TATE positron emission tomography (PET) is octreotide-based and consequently has higher affinity for SSTR2. To identify the most efficient somatostatin analog, a more detailed mapping of the SSTRs expression profile of each individual tumor may be required. This could be conducted with techniques such as immunohistochemistry.

SSTRs are membrane-bound G-protein coupled receptors composed of glycoproteins with seven alpha-helical transmembrane domains. The extracellular N-terminal ensures specific binding of the ligand somatostatin, while the intracellular C-terminal transmits signals through a heterotrimeric G protein consisting of α-, β-, and γ-subunits. This triggers intracellular pathways using guanosine triphosphate (GTP), leading to inhibition of adenylyl cyclase, activation of phosphotyrosine phosphatase (PTP) and modulation of mitogen-activated protein kinase (MAPK), which induces cell cycle arrest [15,16,17,18]. Only SSTR3 may induce PTP-dependent apoptosis followed by activation of p53 and Bax, a pro-apoptotic protein [15,18]. As described, apoptosis and cell cycle arrest may be mediated directly by SSTRs being present on tumor cells, such as meningioma cells. However, effects may possibly also be achieved indirectly by SSTRs present on normal cells. This is accomplished by promotion of vasoconstriction, inhibition of angiogenesis, modulation of immune cell function and decreased secretion of growth factors [15,16,18].

### 3.6. Efficiacy of Somatostatin Analogs

The ten-year relative survival rate for non-malignant meningiomas is 83.4% with age as an important variable, according to CBTRUS. In comparison, the relative survival rate for malignant (CNS WHO grade 3) meningiomas was 60% for all patients, and only 38.5% for patients over 75+ years old [5]. Nevertheless, these numbers include all patients with meningioma in the United States of America and may not provide representative numbers for the prognosis of patients with treatment-refractory meningiomas. To evaluate the efficacy of new drugs, one first must decide on the desired treatment response. Kaley et al recommend benchmarks of PFS-6 of 29% for CNS WHO grade 1 meningiomas and PFS-6 of 26% for CNS WHO grade 2 and 3 meningiomas in clinical trials. These benchmarks are based on the weighted average of progression free survival in studies published on various systemic treatment in surgery- and radiation-refractory meningiomas [49]. Several of the included studies use PFS-6 as a measure of treatment response, and most included studies presented PFS-6 values superior to the stated benchmarks [35,36,38,39,40], yet, some still report the treatment as unsuccessful. One such paper is published by Simó et al, where the radiological partial response (RPR) is set as the primary endpoint and PFS-6 as the secondary endpoint. None of the patients had RPR and PFS-6 of 44.4% is referred to as modest. The same authors also state the challenges of the partly unknown progression of untreated meningiomas, suggesting this as a limitation for clinical research [39]. Another issue, presented by Norden et al, is the absence of larger datasets for comparison [38]. The missing consensus on endpoints troubles the comparisons between studies. Also, standardized image protocols at regular intervals are needed for true assessment of progression free survival. Although radiological response may be a useful endpoint, unsystematic imaging and odd follow-up intervals limit radiological assessment, not at least in retrospective studies. Further on, somatostatin analogs are suggested as effective in prevention of cell proliferation, but not as inducers of cell apoptosis [43]. Hence, somatostatin analogs may be effective in prevention of further tumor growth but may not induce the apoptosis necessary to reduce the existing tumor mass. This could represent a potential limitation for radiological endpoints, at least if primarily looking for radiological responses.

Combination therapy with a somatostatin analog and everolimus, a mammalian target of rapamycin (mTOR) inhibitor, is described by several of the studies, both in vivo [33,35,40] and in vitro [41,42]. In vitro, octreotide and pasireotide were found to enhance the effect of everolimus on decreasing cell viability and proliferation [41,42]. Pasireotide had the most favorable effect [42]. Everolimus inhibits mTOR, which is a serine/threonine protein kinase that regulates growth through general protein biosynthesis. The mRNA translation that encodes proteins that are necessary for S-phase initiation and G1 cell-cycle progression are controlled by the mTOR pathway. As a result, inhibition of the mTOR pathway, using drugs such as everolimus, may result in cell arrest in G1 phase or a prolonged G1 phase. The mTOR pathway serve as a gatekeeper, ensuring G1 phase progression only occur under nutrient-replete conditions [50,51]. Everolimus is currently approved by the United States FDA for treatment of adult patients with progressive, non- functional and well-differentiated neuroendocrine tumors of lung or gastrointestinal origin with locally advanced, unresectable or metastatic disease [52].

As for further clinical use, combination therapy may be an option for heavily pre-treated patients with meningioma [35], and just as prosperous as Sunitinib, a multi-targeted receptor tyrosine kinase inhibitor [33].

### 3.7. Somatostatin Analogs and Previous Treatment

Most of the included patients had already undergone substantial treatment for their meningioma, including surgery and/or radiation [31,35,39]. Hence, their meningioma may be defined as treatment-refractory as the tumor does not respond to treatment. There is no consensus on how to define treatment-refractory meningiomas, and the included studies present a heterogenous group of patients in terms of variables such as age, prior treatments, time since treatments, and comorbidity. As a result, and without adequate control groups, the efficacy of somatostatin analogs might prove difficult to evaluate. As mentioned, responses may not be expected since somatostatin analogs do not induce apoptosis. Thus, slowing or halting growth may perhaps be what we can hope for. Prior treatments could be confounding factors when evaluating both treatment responses and life expectancy following treatment with somatostatin analogs. Further on, several studies only included patients with a Karnofsky status above a pre-decided limit. Patients with higher Karnofsky status may have an overall better ability to tolerate treatment with somatostatin analogs and, hence, also suffer from less adverse effects. On the contrary, other studies only included terminally ill patients with short life expectancy. Both situations may result in selection bias potentially limiting the external validity of results. Any co-medication is poorly described in most studies and could influence clinical results. For instance, patients included in the study conducted by Chamberlain et al could receive dexamethasone, a glucocorticoid, for control of any neurologic symptoms [36]. The duration of treatment with somatostatin analogs is another unexplored factor. For pituitary macroadenomas causing acromegaly, six months of treatment result in treatment responses in approximately 2/3 [53], but optimal treatment algorithms may be very different in meningiomas.

Also, somatostatin analog treatment has so far been used experimentally in treatment-refractory tumors. To be remembered, other effective and established treatments have failed in this situation and expecting a response to drugs in this treatment-refractory state may be unfair or at least less likely. Efficacy could potentially be easier to detect in treatment naïve settings. As the incidence of incidental asymptomatic meningiomas increase, active surveillance, also known as “wait and see”-strategies, is increasingly used for these patients, as treatment, such as surgery, may at times impose more severe complications than the tumor itself [3,10]. Still, the IMPASSE study found stereotactic radiosurgery superior to active surveillance in offering tumor control without risking short term neurological deficits in asymptomatic patients [3]. As a supplement to active surveillance or stereotactic radiosurgery, the potential anti-proliferative effect of somatostatin analogs might be exploited in prevention of further tumor growth.

### 3.8. Side Effects

Side effects of octreotide are described as modest or absent in the included studies. The most frequently reported side effects were abdominal pain and diarrhea [29,37,39,40]. This might be explained by the regulatory role of somatostatin in the endo- and exocrine pancreas, and the gastrointestinal tract. Somatostatin are synthesized and released by nerve cells and endocrine cells in pancreas and the gastrointestinal tract, where the peptide acts paracrine, autocrine or neuronal to inhibit smooth-muscle contractility, glandular secretion, absorption of nutrients, neurotransmission and activated immune cells [54]. Despite often continuous treatment, most symptoms are resolved spontaneously within two weeks as normal organs rapidly adjust their SSTR2 levels and hereby prevent side effects [23,54]. Yet, one case report described autoimmune mediated focal demyelination after treatment with octreotide [32]. Still, octreotide is known as well-tolerated for other diseases, such as acromegaly and gastroenteropancreatic neuroendocrine tumors [23].

### 3.9. Theranostics Utilizing SSTRs

In recent years, theranostics utilizing SSTRs have made its marks in neuro-oncology, including meningiomas, and have recently been recognized by EANO [7]. By using peptide receptor radionuclide therapy (PRRP) with 177Lu-DOTATATE or 90Y-DOTATOC, imaging and therapy is combined, as one radionuclide emits positrons or photons suitable for imaging, while the other emits particles for anti-tumoral effect [55]. Although there are promising preliminary reports for treatment-refractory meningiomas, the efficacy of PRRP is still much unexplored [55,56,57,58,59,60,61]. Furthermore, several issues need resolving, such as the number of cycles, intervals between the cycles and the optimal activity to be administered [55]. According to the Norwegian guidelines for meningiomas, some PET-protocols have demonstrated a high sensitivity in detecting meningiomas, though, the diagnostic and clinical use is still limited. Still, the guidelines underline a potential use for PET scans in atypical and malignant meningiomas, and in relation to targeted radiotherapy [25]. Theranostics utilizing SSTRs is not mentioned in the Norwegian guidelines.

### 3.10. Strenghts and Limitations

Critical appraisal is the systematic evaluation of scientific research to judge its value, trustworthiness, and relevance in a specific context. This systematic scoping review used validated checklists from JBI as a tool for critical appraisal. One question from the critical appraisal checklist is the similarity of the included study participants, which is a question of definition. The studies often included all three CNS WHO grades, yet the study populations still presented with similarities of mostly having treatment-refractory meningiomas with short life-expectancy, regardless of their CNS WHO grade. To be remembered, the effect of any intervention, including the established treatment modalities may be greatly underestimated if only treatment-refractory patients are studied. All studies are non-randomized without control groups and include few patients. Non-randomization presents a clear risk for confounding bias. Retrospective and/or multicenter studies had less standardization in treatment (duration and dosage), imaging protocols and clinical management algorithms. Unexplored and non-controlled variables could present latent bias. Overall, the included studies are of low quality and have a substantial risk of bias. This is also supported by a recently published review on somatostatin analogs in treatment-refractory meningiomas [62].

There is no published randomized clinical trial (RCT) on treatment with somatostatin analogs for patients with meningioma. RCT is the gold standard for drug trials and the absence of a RCT study presents a substantial missing piece for the knowledge on somatostatin analogs in meningiomas. As for limitations of this systematic scoping review, two independent reviewers for the inclusion process could have been advisable. However, precise exclusion and inclusion criteria were set to ensure reproducibility.

## 4. Conclusions

In conclusion, various efficacy of somatostatin analogs is reported by the included studies. None of the studies are randomized or controlled, and the overall quality of evidence is low. Further, the lack of standardized endpoints, imaging protocols and heterogenous case selection, and variation in drugs and doses limit the comparison of results across studies. In any case, the reported side effects of somatostatin analogs are sparse and well-known from other patient groups. Given the possible effect observed in some studies, somatostatin analogs may present a safe last-option treatment in severely ill patients with treatment-refractory meningiomas. However, only a properly controlled study, preferably a RCT study could clarify the efficacy on somatostatin analogs. Also, detection of potential treatment effects may perhaps be easier if done in a treatment-naïve and not a treatment-refractory clinical setting.

## Figures and Tables

**Table 1:** Overview of the 13 included in vivo papers.

Author, Year, Location [Ref]	Number of Patients	CNS WHO Grade and/or Subtype	Treatment	Clinical Response, PFS-6	Radiological Response	SSTR Status	Adverse Effects
**Case Reports (*n* = 5)**							
Buigues, 2016, Spain [28]	1	Meningothelial meningioma	Lanreotide sc	None, PFS > 2 years	Improvement	OctreoScan positive	Not reported
García-Luna, 1993, Spain [29]	3	Meningothelial and papillomatous meningioma	Octreotide sc	No lasting response	None	SSTRs confirmed in one case, otherwise unknown	None/mild adverse effects
Jaffrain-Rea, 1998, Italy [30]	1	Transitional meningioma	Octreotide sc	Clinical improvement	None	Not reported	Not reported
Rammo, 2016, USA [31]	1	Grade 3	Octreotide	Remission for 3.5 years	Not reported	Octreotide receptor 2a present	Not reported
Schreglmann, 2013, Switzerland [32]	1	WHO grade 1, meningotheliomatous, Pulmonary metastases	Octreotide sc	Not reported	New tumor lesions	Octreotide scintigraphy positive	Autoimmune-mediated focal demyelination. Polyallergic patient
**Prospective Studies (*n* = 5)**							
Chamberlain, 2007, USA [36]	16	WHO grade 1, 2, and 3	Sandostatin LAR im	PFS-6 *: 44%	Partial radiological response: 31% of patients	Octreotide scintigraphy positive	Minimal toxicity
Graillon, 2020, France [40]	20	WHO grade 1, 2, and 3	Everolimus po + octreotide LAR im	PFS-6: 55%	Anti-tumor activity (3D tumor growth rate)	SSTR2a detected in most patients using IHC	Stomatitis in 55% of patients, discontinuations of two patients
Johnson, 2011, USA [37]	11	WHO grade 1, 2, and 3	Octreotide sc	None	None	SSTR status partly known	Mild adverse effects: diarrhea, nausea, anorexia, transaminitis
Norden, 2015, USA [38]	34	WHO grade 1, 2, and 3	Pasireotide LAR im	WHO grade 1: 50% WHO grade 2 + 3: 17%	None	High octreotide uptake	Treatment well tolerated
Simó, 2014, Spain [39]	9	WHO grade 2 and 3	Octreotide im	PFS-6: 44.4%	None	Positive octreotide SPECT scanning	Minimal toxicity reported
**Retrospective Studies (*n* = 3)**							
Cardona, 2019, Colombia [33]	15 received octreotide	WHO grade 2 or 3	Octreotide ± everolimus	No difference in survival when comparing everolimus ± octreotide, and sunitinib	Not reported	Confirmed overexpression of SSTR2	Fatigue and oedema
Furtner, 2016, Austria [34]	9 received somatostatin analog	WHO grade 2 and 3	Somatostatin analog	Not reported	No reduction in peritumoral edema or tumor size	SSTR status not reported	Not reported
Le Van, 2021, France [35]	8	WHO grade 1, 2, and 3	Everolimus + octreotide	PFS-6 *: 60%, combination of octreotide + everolimus	Not reported	SSTR status not reported	Not reported

* Progression-Free Survival at 6 months. Abbreviations: subcutaneous (sc), intramuscular (im), per oral (po), long-acting release (LAR), immunohistochemistry (IHC).

**Table 2:** Overview of the four included in vitro investigations.

Author, Year, Location [Ref]	Aims	Results
Graillon, 2015, France [41]	Activity of octreotide, everolimus, BKM-120 and BEZ-235 (new Pi3K/Akt/mTOR inhibitors), and a combined treatment (octreotide plus everolimus) on signaling pathways, cell proliferation, and cell cycle proteins in meningioma primary cells.	*n* = 23 patients. SSTR2 mRNA expression in all tested cells. Octreotide decreased cell viability. Enhanced decrease with a combined treatment of octreotide and everolimus.
Graillon, 2017a, France [42]	Comparison of pasireotide and octreotide, both alone and in combination with everolimus, on meningioma primary cell cultures.	Pasireotide induces a higher reduction in cell viability and stronger inhibitory effect on cell proliferation than octreotide, both alone and in combination with everolimus.
Graillon, 2017b, France [43]	Evaluate the effect of octreotide on meningioma primary cell cultures.	*n* = 80 meningioma primary cell cultures. Octreotide significantly decreased cell proliferation in the majority of meningiomas but did not induce apoptosis. Improved octreotide effect on cell viability if elevated level of SSTR2.
Koper, 1992, Netherlands [44]	Effects of somatostatin and octreotide on the growth of cultured human meningioma cells.	Significant stimulation of growth.

## Data Availability

Not applicable.

## Supplementary Materials

The following supporting information can be downloaded at: https://www.mdpi.com/article/10.3390/ijms24054793/s1.
