# Supplementary material for: Meningiomas and Somatostatin Analogs: A Systematic Scoping Review on Current Insights and Future Perspectives
Source: Int J Mol Sci. 2023 Mar 1;24(5):4793. doi: 10.3390/ijms24054793 (PMC10003463; doi:10.3390/ijms24054793)
Supplement: Supplementary file 1 [file ijms-24-04793-s001.zip › ijms-2131640-supplementary.pdf]

Supplementary File S1:

Specific search strategies used for the different search databases

PubMed

#	Search	Results
1	Meningioma [Mesh]	20,947
2	Meningioma*	28,730
3	1 OR 2	28,730
4	“somatostatin analogue*”	4,095
5	“somatostatin analog*”	9,379
6	“somatostatin receptor agonist*”	69
7	octreotide	11,334
8	sandostatin	11,439
9	lanreotide	1,317
10	pasireotide	678
11	Octreotide [Mesh]	8,112
12	Lanreotide [supplementary concept]	849
13	Pasireotide [supplementary concept]	421
14	angiopeptin	1,337
15	Angiopeptin [supplementary concept]	849
16	4 OR 5 OR 6 OR 7 OR 8 OR 9 OR 10 OR 11 OR 12 OR 13 OR 14 OR 15	16,853
17	3 AND 16	219

Embase via Ovid

#	Search	Results
1	exp meningioma/	31109
2	meningioma.ti,ab,kw.	21216
3	meningiomas.ti,ab,kw.	15453
4	1 or 2 or 3	35793
5	exp octreotide/ or somatostatin derivative/	27833
6	octreotide.ti,ab,kw	12719
7	sandostatin.ti,ab,kw.	914
8	"somatostatin analogs".ti,ab,kw.	2803
9	"somatostatin analog".ti,ab,kw.	2221
10	"somatostatin analogue".ti,ab,kw.	3144
11	"somatostatin analogues".ti,ab,kw.	3448
12	"somatostatin receptor agonists".ti,ab,kw.	51
13	"somatostatin receptor agonist".ti,ab,kw.	37
14	exp angiopeptin/ or somatostatin derivative/	10793
15	lanreotide.ti,ab,kw.	1712
16	exp pasireotide/	1701
17	pasireotide.ti,ab,kw.	888
18	5 or 6 or 7 or 8 or 9 or 10 or 11 or 12 or 13 or 14 or 15 or 16 or 17	33324
19	4 and 18	381

Web of Science:

#	Searches	Results
1	TS=meningioma*<br><br>Indexes=SCI-EXPANDED, SSCI, A&HCI, CPCI-S, CPCI-SSH, ESCI Timespan=All years	22,739
2	TS="somatostatin derivate" OR TS="somatostatin analog*" OR TS="somatostatin analogue*" OR TS="somatostatin receptor agonist*" OR TS=octreotide OR TS=sandostatin OR TS=lanreotide OR TS= pasireotide OR TS=angiopeptin<br><br>Indexes=SCI-EXPANDED, SSCI, A&HCI, CPCI-S, CPCI-SSH, ESCI Timespan=All years	17,700
3	#2 AND #1<br><br>Indexes=SCI-EXPANDED, SSCI, A&HCI, CPCI-S, CPCI-SSH, ESCI Timespan=All years	200
